# Neurophysiological and Behavioral Differences between Older and Younger Adults When Processing Violations of Tonal Structure in Music

**DOI:** 10.3389/fnins.2018.00054

**Published:** 2018-02-13

**Authors:** Marie-Élaine Lagrois, Isabelle Peretz, Benjamin Rich Zendel

**Affiliations:** ^1^International Laboratory for Brain, Music, and Sound Research, Montréal, QC, Canada; ^2^Département de Psychologie, Université de Montréal, Montréal, QC, Canada; ^3^Faculty of Medicine, Memorial University of Newfoundland, St. John's, NL, Canada

**Keywords:** aging, music, event-related potentials, attention, ERAN, P600

## Abstract

Aging is associated with decline in both cognitive and auditory abilities. However, evidence suggests that music perception is relatively spared, despite relying on auditory and cognitive abilities that tend to decline with age. It is therefore likely that older adults engage compensatory mechanisms which should be evident in the underlying functional neurophysiology related to processing music. In other words, the perception of musical structure would be similar or enhanced in older compared to younger adults, while the underlying functional neurophysiology would be different. The present study aimed to compare the electrophysiological brain responses of younger and older adults to melodic incongruities during a passive and active listening task. Older and younger adults had a similar ability to detect an out-of-tune incongruity (i.e., non-chromatic), while the amplitudes of the ERAN and P600 were reduced in older adults compared to younger adults. On the other hand, out-of-key incongruities (i.e., non-diatonic), were better detected by older adults compared to younger adults, while the ERAN and P600 were comparable between the two age groups. This pattern of results indicates that perception of tonal structure is preserved in older adults, despite age-related neurophysiological changes in how melodic violations are processed.

## Introduction

Age-related difficulties with hearing are due to changes in physical structures of the inner ear, as well as changes to central processing of incoming acoustic information (Gates and Mills, 2005; Tun et al., 2012; Profant et al., 2015). These changes lead to myriad hearing difficulties, the most common being a difficulty understanding speech, particularly in noisy environments (Schneider et al., 2010; Ouda et al., 2015). Speech perception has been extensively studied in older adults, using both behavioral and neuroimaging techniques (e.g., Pichora-Fuller et al., 1995; Wong et al., 2009). In contradistinction to speech perception, little is known about how processing of music changes with age, particularly at the neurophysiological level. Still, there is some evidence that music perception abilities are relatively preserved in older adults (Halpern et al., 1995; Halpern and Bartlett, 2002). It is therefore likely that the underlying neural processing of music changes with age, in order to compensate for age-related decline in the auditory domain. These putative neurophysiological changes to music processing may also provide clues to how the brain adapts to other age-related changes in cognition, which could be useful for developing evidence-based cognitive rehabilitation programs for older adults. Accordingly, the goal of the current study was to determine if there are differences in the perception of tonal structure between older (60+ years) and younger adults (<35 years) using both behavioral and electrophysiological measurements.

In the musical domain, tonal structure is one critical aspect of music perception. The Western tonal system is composed of twelve tones, each interleaved by one semitone, forming the chromatic scale. From those tones, seven tones are taken to form the diatonic scales, like the major and minor scales (ex: do re me fa so la ti do). Melodies from the Western tonal system are usually based around diatonic scales, in a specific key (e.g., C major, A minor). Additionally, statistical regularities guide the relationships between a note and the preceding notes of a given diatonic context. These regularities allow listeners to acquire implicit knowledge of tonality by passive exposure to music throughout life (Tillmann et al., 2000). Importantly, knowledge of tonal structure does not require formal training, as healthy adults with no musical training will rate the belongingness of a note presented in a tonal context in a manner consistent with Western music theory (Cuddy and Badertscher, 1987). Specifically, through statistical learning, listeners are able to predict the probability of hearing an upcoming tone in a melody (Pearce and Wiggins, 2012). Accordingly, greater exposure to the rules of the tonal system throughout life would endow the listener with stronger tonal expectancies.

Evidence suggests that tonal knowledge is spared with aging, as older adults are generally able to detect tonal violations as well as younger adults (Halpern et al., 1995, 1996, 1998). However, these studies used melodic similarity judgments or the probe tone paradigm, where listeners rated the belongingness of a probe tone after hearing a tonal context. These kinds of studies provide a good indication that tonal knowledge is preserved in older adults but give little information on tonal processing. Other studies have demonstrated that, when making a tonal judgment, older adults remained slightly more susceptible to the impact of irrelevant auditory information like pitch height or stimulus duration, consistent with a decline of attentional control or inhibitory processing with aging (Halpern et al., 1995; Alain and Woods, 1999). This points to the idea that although tonal knowledge might be preserved in older adults, there are likely general cognitive changes occurring that can impact how tonal structure is processed. These changes would likely be reflected at the neurophysiological level. In that respect, the event-related potential (ERP) technique could give us a window toward a better comprehension of how aging interacts with cognition, and tonal knowledge, in the perception of tonal structure.

One way to assess the intersection of tonal processing and cognition is to use a paradigm developed by Brattico et al. (2006). In this paradigm, ERPs were recorded while participants were presented with melodies that sometimes contained a tonal violation. During a passive condition, participants were told to ignore the melodies and watch a silent film with subtitles. In this condition, attention directed toward the tonal violation was minimized in order to focus on automatic, stimulus-driven, responses to tonal violations. In this context, the neurophysiological response to a tonal violation was highlighted by an increase in the ERPs negativity for the tonal violation. This response occurred between 150–250 ms after the onset of the violation over right frontal electrodes and could be referred to as an early right anterior negativity (ERAN), although the authors call it a MMN. The ERAN is thought to reflect the automatic detection of a syntactical violation in a melodic context, and this is usually a chord or a note that does not fit the key of the melody or the harmony (Koelsch et al., 2007).

In an active condition, participants were asked to listen to each melody and rate how congruous the melody was on a 7-point scale. This condition required attention and therefore cognitive/controlled processing of the tonal violation. In the active condition, a second neurophysiological response was evoked by the tonal violation and was highlighted by an increase in the ERPs positivity. This response, called a P600, occurred around 500–700 ms after the onset of the violation and was widespread over the scalp. The P600 is comparable to P3-type responses, in that these responses are normally evoked by the awareness of a target stimulus (Polich, 2007). The P600 is, however, more specifically related to violation of syntax in a melodic context (Besson and Faïta, 1995; Patel et al., 1998; Brattico et al., 2006) or a speech context (e.g., Osterhout and Holcomb, 1993; Hahne and Friederici, 1999). In both conditions, Brattico et al. (2006) used two kinds of tonal violations, which belonged to two different levels of tonal syntax. The lowest level violation corresponded to a note that was non-chromatic (i.e., not a note in Western music). This type of violation was referred to as an out-of-tune note. The second level violation corresponded to a violation of the diatonic scale. A non-diatonic note would be a note that is from the chromatic scale, but not part of the scale used to construct the melody. This type of violation was referred to as an out-of-key note. Brattico et al. (2006), showed that both types of tonal violations evoked an ERAN and P600 type response. Furthermore, out-of-tune notes were more salient than out-of-key notes, as participants could more easily detect out-of-tune notes and the ERAN evoked by these notes was larger. This finding was consistent with the idea that an out-of-tune note violates a lower level of tonal organization, that is, the basic knowledge of the semitone interval governing the organization of the Western tonal system. On the other hand, detection of the out-of-key note would require specific knowledge of the diatonic scale. This type of paradigm and nomenclature has been used in a number of studies that investigated melodic processing while recording electrical brain responses (e.g., Trainor et al., 2001; Peretz et al., 2009; Zendel et al., 2015).

The distinction between hierarchical levels in tonal organization was of particular interest for the current study. Indeed, comparing how younger and older adults can detect both out-of-tune and out-of-key notes can help determine the interplay between automatic and cognitive/controlled processing with aging by examining how the ability to detect tonal violations interacts with the ERAN and P600 differently in older and younger adults. As one ages, one would be exposed to more music, and thus statistically driven representations of diatonic tonal structure would be enhanced. This could confer an advantage to older adults for the detection of out-of-key notes, compared to out-of-tune notes. To our knowledge, no study has yet directly compared an automatic and controlled responses to music syntactic violations in older adults, using both a passive and active listening task.

A number of studies have examined how aging impacts ERPs to other types of auditory stimuli. One of the most well studied responses is the mismatch negativity (MMN), a lower-level process generally associated with sensory memory (Näätänen et al., 2007; Koelsch and Jentschke, 2009; Kalda and Minati, 2012). This response occurs during a similar time frame as the ERAN and shares the general property of representing a violation of auditory expectancy; however, there are differences between these responses (see Koelsch, 2009). In aging, the MMN can reflect age-related deficits in auditory discrimination (Alain et al., 2004; Cooper et al., 2006; Rimmele et al., 2012; Cheng et al., 2013) and in auditory short-term memory (Näätänen et al., 2012). However, age-related differences in MMN are not always found (Bellis et al., 2000; Fabiani et al., 2006). Interestingly, the studies that reported an age effect on the MMN used pure-tone stimuli, while the studies that did not report an age effect on the MMN used more complex stimuli [e.g., speech sounds (Bellis et al., 2000) or harmonic complex (Fabiani et al., 2006)]. This suggests that the age-related differences are reduced when the stimuli are more harmonically complex. Given that music is more complex than pure-tone sequences, it is possible that there will be no age-related difference on the ERAN.

Only one study has investigated the P600 evoked by melodic expectancy in older adults. Halpern et al. (2017) found that unexpected endings in melodies elicited a P600-type response of similar amplitude in younger and older adults over parietal sites, although the P600 was more broadly distributed over the scalp in older adults. The P600 component has also been measured in older adults in response to speech syntax violations (Kemmer et al., 2004; Steinhauer et al., 2010). These violations evoked a P600 response of comparable amplitude in older and younger adults, although the response was more anterior in older adults. These results highlight a potential compensatory mechanism for detecting syntactic violations in speech or music, as an anterior shift could potentially be due to an enhanced contribution of frontal generators of the P600. Additional studies have investigated other late positive responses to violations of expectancy in the auditory domain. These P3 responses are typically smaller and delayed in older adults (Anderer et al., 1996; Alain et al., 2004; Schiff et al., 2008).

The goal of the current study was to compare the ability to detect tonal violations and the associated neurophysiological responses between older and younger adults. The experimental design was a modified version of the protocol used by Brattico et al. (2006). The main modification was to use a click-detection task instead of a passive-listening task to better control the deployment of attention away from the tonal structure of the melody (see Method). We expected that the response to out-of-key notes would be enhanced in older adults. This should be paralleled by a comparable ERAN when attention was directed away from the melody, and an enhanced P600 when actively detecting the tonal violation. This would imply a more robust representation of the diatonic scales in older adults. In contrast, the ability to detect an out-of-tune note and the associated brain responses in older adults should be similar relative to younger adults. This would indicate that the representation of the chromatic scale reaches a maximum early in life, and due to constant exposure to the intervals in the chromatic scale, is preserved in older adults.

## Methods

### Participants

Sixteen adults were recruited in the younger group via an online advertising platform and advertisement on the university campus. Of these, one participant was excluded because he did not press one of the response buttons for more than twenty percent of the trials and three participants were excluded because of a noisy EEG signal on more than half the trials for at least one experimental condition. The 12 remaining young participants were aged from 18- to 35-years-old (mean = 25.7, *SD* = 6.3; 6 females). The older group was taking part in a parallel study in our lab at the same time period, as a control group for older people with amusia (music processing deficiency; Zendel et al., 2015). This group was composed of eleven participants aged between 59- and 73-years-old (mean = 64.3, *SD* = 4.3; 8 females). The age of the participants differed significantly between groups, *t*_(21)_ = 17.04, *p* < 0.001. There was no significant difference between groups regarding years of education (Young adults: mean = 15.8, *SD* = 2.7; Older adults: mean = 15.5, *SD* = 2.3; *t*_(20)_ = 0.34, *p* = 0.74; note that data from one participant in the younger group was missing). All participants were right-handed and reported no hearing impairment nor neurological disorder. Due to the nature of the group comparison, pure-tone thresholds were only collected for the older adults. The pure-tone average was calculated (PTA; 0.5, 1, 2 kHz in both ears). Three older participants had PTA thresholds between 25–40 dB HL, indicating mild hearing impairment; all others had thresholds below 25 dB HL (i.e., normal hearing; mean = 18.4, *SD* = 11.9). This range of PTA thresholds is normal in a sample of older adults. Stimuli in the study were presented well above threshold, and no participants had difficulty hearing the stimuli. The participants in this study did not report having specific difficulties with music. All participants had <5 years of formal musical training (Young adults: mean = 0.1, *SD* = 0.3; Older adults: mean = 0.7, *SD* = 1.2). There was no significant difference between groups on years of musical training, *t*_(21)_ = 1.75, *p* = 0.11. All subjects gave written informed consent in accordance with the Declaration of Helsinki and received monetary compensation for their participation. All procedures were approved by the Research Ethics Council for the Faculty of Arts and Sciences at the Université de Montréal.

### Stimuli

**Figure 1:**
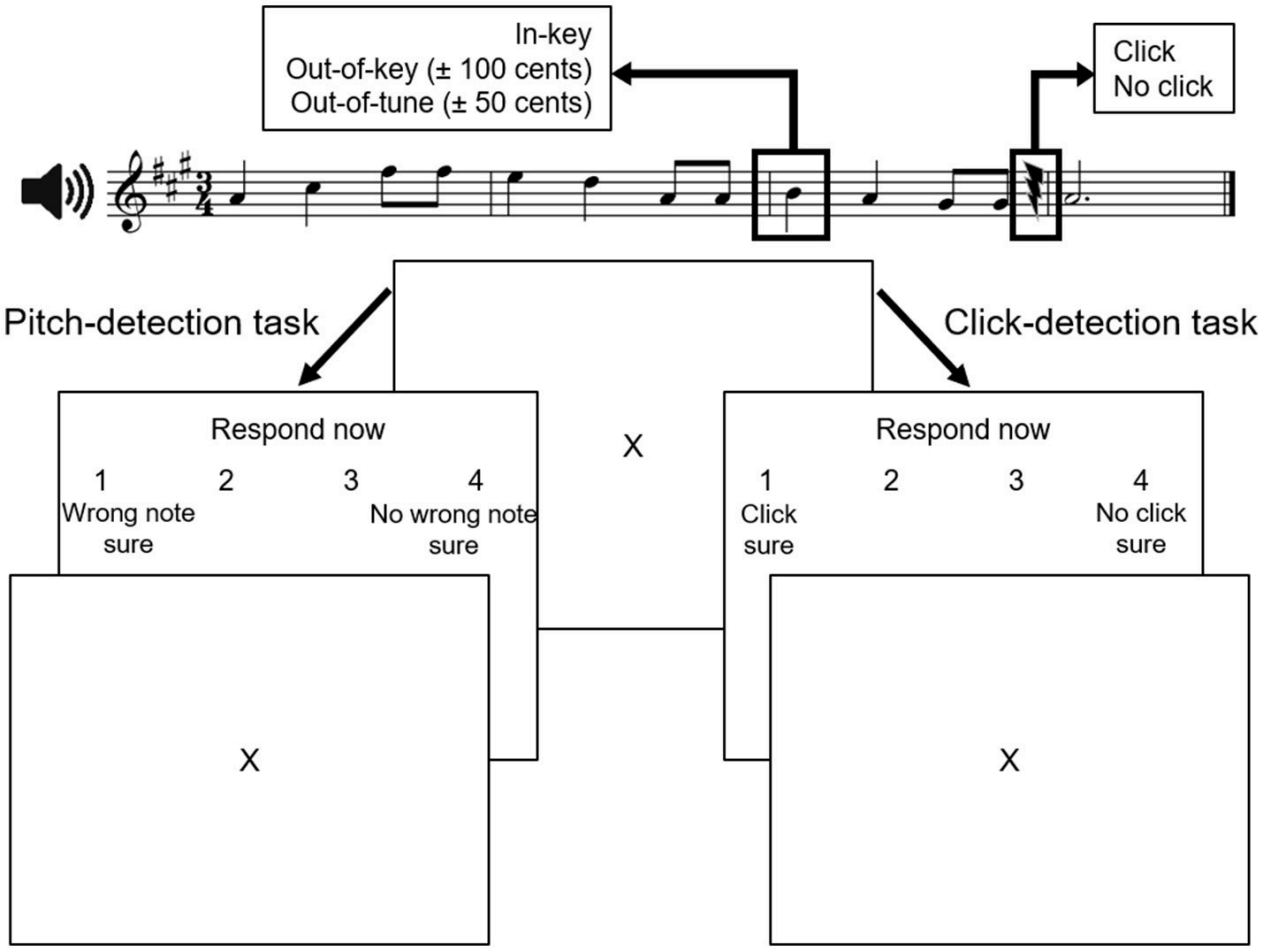
Experimental paradigm (adapted from Zendel et al., 2015). Stimuli in both tasks were similar. During the pitch-detection task, participants were asked to identify whether the melody contained a wrong note. During the click-detection task, participants were asked to identify if they heard a near-threshold click in the melody. The two tasks were completed in separate blocks of trials.

Participants were presented with 40 novel melodies constructed from the Western major scale. On average, melodies had 10.3 notes (range: 7–15 notes) and lasted 5.4 s (range: 2.8–12 s). They were randomly mixed with the same melodies in which 40 target tones were played out-of-key [±100 cents (1 semitone)] and 40 target tones that were out-of-tune [±50 cents (one-half semitone)]. The target-tone was always on the first beat of the third bar, and was 500 ms in duration. Clicks were inserted in half the melodies. When present, the click occurred at least two notes after the target tone, and was calibrated in amplitude such that an individual listener could detect ~75% of the clicks. Accordingly, there were six versions of each melody (i.e., in-tune, click; in-tune, no click; out-of-tune, click; out-of-tune, no click; out-of-key, click; out-of-key, no click) yielding a total of 240 melodies. For all participants, two distinct sets of 120 stimuli, comprising three randomly mixed versions of each melody, were used in each task. In each task, each type of target tone (in-key, out-of-tune, or out-of-key) occurred in one-third of the melodies and half of the melodies contained a click. A sample stimulus is illustrated at the top of Figure 1.

### Procedure

Data recording took place in a faradized and sound-isolated room during a single session, lasting about 3 hours. Melodies were presented binaurally through Etymotic (ER-2) insert earphones at 75 dB SPL. The interstimulus interval was of 2270 ms, including the response time window of 2000 ms, and a bell ring of 270 ms indicating the beginning of the next trial. Answers were recorded for each trial and electroencephalographic data were collected continuously from the beginning to end of each task.

The procedure was identical to that described in Zendel et al. (2015). Participants first completed the click-detection task. To determine the starting intensity of the click, participants were presented with melodies that contained a click and asked if they could hear the click. The first click was at 76 dB SPL. Then, the intensity of the click was reduced in steps of 10, 5, 5, 3, and 1 dB until the participant could no longer detect the click. The intensity of the click was then increased in 1 dB steps until the participant could hear the click again. The final intensity of the click at the end of the procedure was chosen as the starting click intensity in the click-detection task. In the click-detection task, participants were asked whether they heard a click in the melody and how sure they were that they heard the click. After each melody, participants could respond, “click, sure,” “click, not sure,” “no click, not sure,” or “no click, sure” by pressing a button on a computer keyboard. Participants were not informed that the melodies could contain an out-of-key or out-of-tune note during the click-detection task. To maintain individual accuracy level at ~75% correct, the intensity of the clicks was continuously adjusted during the task. Based on the accuracy of the eight previous trials, the intensity of the click was decreased by 2.25 dB if all eight trials were accurate and by 0.75 dB if the accuracy was seven out of eight. The intensity was increased by 0.75 dB if accuracy was five out of eight, and by 1.5 dB if accuracy was four out of eight or below.

Next, participants completed the pitch-detection task. In this task, participants were asked whether they heard an incongruous note in the melody, and how sure they were that there was an incongruous note. After each melody, participants could respond “wrong note, sure,” “wrong note, not sure,” “no wrong note, not sure,” or “no wrong note, sure” by pressing a button on a computer keyboard. For the pitch-detection task, participants were told to ignore the clicks. For both tasks, the response choices were displayed on the screen (Figure 1) and the position of the response buttons on the keyboard was counterbalanced across participants. Before each test, there were twelve practice trials that included performance feedback. No feedback was provided for the experimental trials. The pitch-detection and click-detection tasks were not counterbalanced, so participants remained blind to the presence of the out-of-key and out-of-tune notes during the click-detection task, allowing for attention to be focused on detecting the click and not on tonal anomalies.

In the study by Brattico et al. (2006), participants watched a silent subtitled film and were instructed to ignore the experimental stimuli (i.e., melodies that could contain a tonal violation). The purpose of this manipulation was to control for the deployment of attention. This allowed the researchers to analyze neurophysiological responses to tonal deviants that occurred automatically, in this case the ERAN (the authors call it a MMN; however, see Koelsch, 2009), and responses that were dependant on attending to the tonal deviant, in this case the P600. This type of manipulation allowed for a dissociation between automatic and controlled (attention-dependant) processes; however, by using a film it remains possible that a participant could direct their attention to the tonal stimuli, and engage in controlled processing of tonal structure. To better control the deployment of attention we choose to have listeners perform a difficult auditory detection task that did not require the listener to attend to the tonal structure of the melody. This does not preclude the possibility that participants may shift their attention during the click-detection task; however, by performing the click-detection task, the ability to deploy attentional resources (i.e., controlled processes) to the tonal structure of the melody would be greatly reduced compared to the pitch-detection task. More importantly, the potential to deploy attention during the click-detection task would be similar for all participants because the difficulty of the click-detection task was calibrated to be similar for each participant. Therefore, while this manipulation may not have eliminated controlled processing of tonal structure during the click-detection task, it would minimize it. Accordingly, we acknowledge that some controlled processing of tonal structure may have occurred during the click-detection task; however, it would be reduced compared to the pitch-detection task. For the sake of clarity, and consistency with previous research we will refer to neurophysiological responses to tonal deviants evoked during the click-detection task as automatic, and those evoked by the pitch-detection task as automatic and controlled.

### EEG recording and data preprocessing

Electric brain activity was digitized continuously from 70 active electrodes at a sampling rate of 256 Hz, with a high-pass filter set at 0.1 Hz, using a Biosemi Active Two system (Biosemi). Five electrodes were placed bilaterally at mastoid, inferior ocular, and lateral ocular sites (M1, M2, IO1, LO1, LO2). All averages were computed using Brain Electrical Source Analysis (BESA; version 6). ERPs were averaged to the onset of the target note (i.e., out-of-tune note, out-of-key note, or in-key note), and the analysis epoch included 200 ms of prestimulus activity and 900 ms of poststimulus activity. Continuous EEG was then averaged separately in the click-detection and pitch-detection task for each Note Type (i.e., in-key, out-of-tune, and out-of-key) and each electrode site.

Prototypical eye blinks and eye movements were extracted from the continuous EEG. A principal component analysis of these averaged recordings provided a set of components that best explained the eye movements. These components were then decomposed into a linear combination along with topographical components that reflected brain activity. This linear combination allowed the scalp projections of the artifact components to be subtracted from the experimental ERPs to minimize ocular contamination, such as blinks and vertical and lateral eye movements, for each individual (Berg and Scherg, 1994). After this correction, trials with >120 μV of activity were considered artifacts and excluded from further analysis. Overall, 14.8% of the trials were rejected, with ANOVA including Age Group, Task and Note Type showing no significant difference for the Note Type, *F*_(1, 21)_ = 1.03, *p* = 0.36. There was however an interaction between Age Group and Task, *F*_(1, 21)_ = 7.01, *p* = 0.015, *n*^2^ = 0.25, as there were more rejected trials in younger adults (mean = 19.7%; SD = 13.5) compared to older adults (mean = 9.4%, SD = 8.1), for the click-detection task, *t*_(21)_ = 2.18, *p* = 0.041. The 200 ms prestimulus interval was used as a baseline. Averaged ERPs were then bandpass filtered to attenuate frequencies < 0.7 Hz and >20 Hz and referenced to the linked mastoid.

### EEG data analysis

To quantify the EEG data, a series of pairwise permutation tests was done using BESA statistics (version 1.0; Maris and Oostenveld, 2007). The analysis was entirely data driven and included every time point at each electrode in the analysis. This analysis proceeds in three steps. In the first step a series of *t*-tests compared each group/condition at every time point and at every electrode. This mimics a more traditional analysis of ERPs; however, it has the advantage of not relying of a preselection of electrodes or epoch(s). When exploring data where the effect may be dynamic over scalp topography or over time, this approach eliminates any possible hypothesis driven bias into the results. When examining differences between older and younger adults, this is critical as the cortical sources of cognitive processing may be different and be delayed. The major disadvantage of this approach is that due to the number of comparisons (number of electrodes × number of samples in ERP) the probability estimates will be inflated, and will therefore increase the likelihood of making a type 2 error. An adjustment to the *p*-value based on the number of comparisons (i.e., Bonferonni) would not be appropriate for two reasons. The first is that the statistical threshold would become too small to identify any real differences, thus inflating the type 1 error rate. The second reason is that each observation is not independent. Effects at adjacent time points and electrodes are likely related to the same underlying effect. To mitigate these major disadvantages, two more steps are taken in this analysis. In a second step spatio-temporal clusters are formed to identify clusters electrodes over time of the same underlying effect. These clusters are formed by grouping electrodes and time points where the initial *t*-test was significant with adjacent electrodes and time points. Accordingly, clusters were dynamic; that is, the electrodes that formed a cluster could change over the identified time frame. Critically, the formation of these clusters was entirely data driven. In the third step, probability estimates were derived by using a nonparametric permutation-based approach. This permutation test involved comparing the clusters identified in the previous step by randomly assigning data into two groups/conditions, and repeating the statistical analysis. In most situations, this should yield a non-significant effect based on the hypothesis that the group/condition had a real effect on the ERPs. If there was no effect of group or condition on the ERPs, and the difference was due to chance, then any random permutation of the data should reveal significant differences. Accordingly, to derive a probability estimate, 1,000 permutations are calculated, and the *p*-value can be derived from the number of these random permutations that are significant (Maris and Oostenveld, 2007). For example, if 40/1000 of the random permutations are significant, the *p*-value would be 0.04; if 800/1000 permutations are significant the *p*-value would be 0.8. The percentage of permutations where the largest *t*-value in the cluster was significant provides an estimate of likelihood of the original difference being due to chance alone (i.e., a *p*-value; Maris and Oostenveld, 2007). All significant clusters are reported by *p*-values; clusters with the lowest *p*-value are reported first.

The first part of the current analysis focused on within-subject effects by comparing the ERPs recorded to the out-of-tune and out-of-key notes to the in-key note in both groups. These comparisons were performed to identify the ERAN and the P600. The ERAN was expected as a difference in the ERP evoked by the out-of-key or out-of-tune note that was more negative than the in-key note during the 100–300 ms epoch at frontocentral electrodes. The P600 was expected as a difference in the ERP evoked by the out-of-key or out-of-tune note that was more positive than the in-key note during the 400–800 ms epoch at posterior electrodes. These epochs were chosen because previous studies have demonstrated that the ERAN (Koelsch, 2009, 2011) and P600 (Besson and Faïta, 1995) occur within these time frames and at these electrode sites. Other differences between the tonal deviants and the in-key note may also be identified by this procedure.

To determine Age Group differences, a second analysis compared the ERAN and P600 between younger and older adults. First, difference waves were calculated separately between the out-of-key/out-of-tune melody and the in-key melody. This isolated the impact of pitch deviance in each participant, and allowed for permutation testing of this effect between groups.

### Behavioral data analysis

Behavioral data were analyzed separately for the pitch-detection and click-detection tasks. The ratings (1–4) were separated into accuracy and confidence scores. A trial was considered accurate if the person made the correct judgment, regardless of his or her confidence. If correct, it was scored as 1; if incorrect, it was scored as 0. Raw accuracy was the overall percentage of correct responses. For group comparisons, accuracy was further calculated as hits minus false alarms (H-FAs). In the click-detection task, a false alarm corresponded to the hearing of a click when there was none. Similarly, in the pitch-detection task, a false alarm corresponded to reporting a wrong note when there was none. The H-FA scores were calculated separately for in-key (click-detection task only), out-of-key, and out-of-tune melodies. H-FAs could not be calculated for the in-key note during the pitch-detection task because responses to the in-key note were needed to calculate the false alarm rate for out-of-key and out-of-tune notes. Confidence was quantified by separating “sure” from “not sure” responses regardless of the judgment. “Sure” responses were coded as 1. “Not sure” responses were coded as 0. Confidence was the percentage of trials reported as “sure.” These responses were analyzed using mixed-design ANOVAs that included Age Group (younger, older) and Note Type [in-key (click-detection task only), out-of-key, out-of-tune].

## Results

### Behavioral results

#### Click-detection task

**Figure 2:**
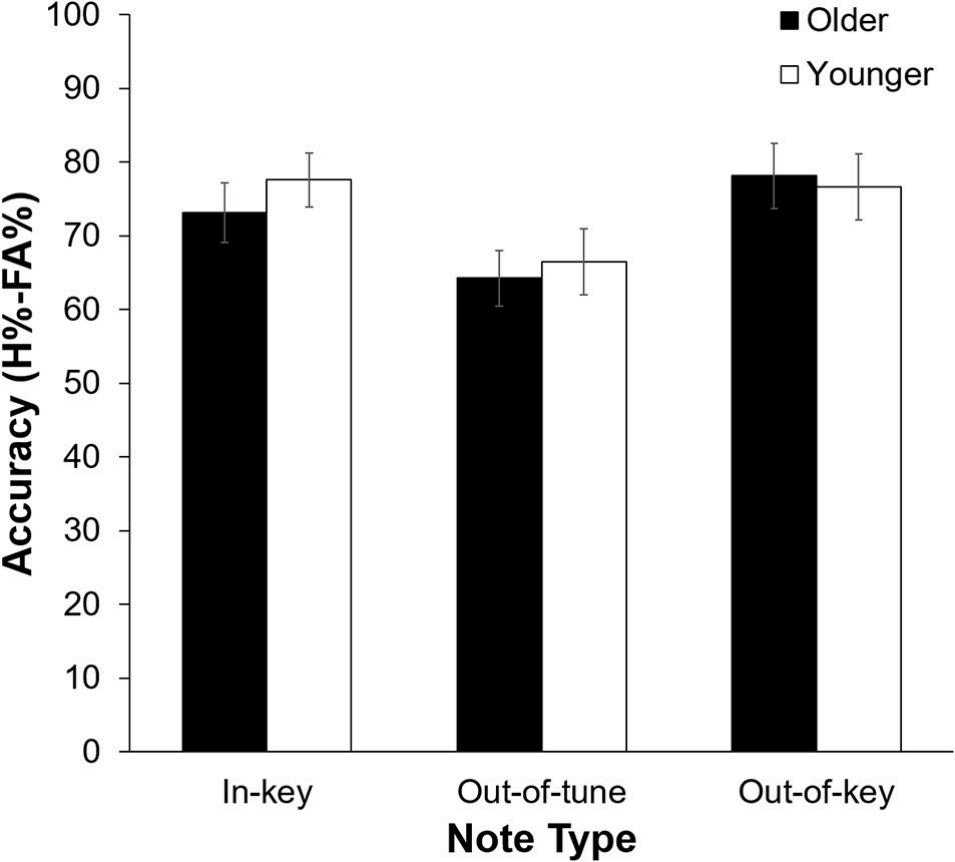
Click-detection accuracy (H%–FA%) as a function of Age Group, and Note Type. Overall, accuracy was lower when the click was preceded by an out-of-tune note compared to when the click was preceded by an in-key or out-of-key note (*p* < 0.001 for both). Errors bars represent one standard error of the mean.

As can be seen in Figure 2, pitch deviance had an impact on click-detection accuracy, *F*_(2, 42)_ = 12.60, *p* < 0.001, *n*^2^ = 0.375. Pairwise comparisons revealed that click-detection accuracy was lower after an out-of-tune note compared to both an out-of-key note, *t*_(22)_ = 4.23, *p* < 0.001, and an in-key note, *t*_(22)_ = 4.67, *p* < 0.001. The main effect of Age Group, *F*_(1, 21)_ = 0.11, *p* = 0.74, and its interaction with Note Type, *F*_(1, 21)_ = 0.68, *p* = 0.51, were not significant. Confidence was impacted by the presence of a click, with participants being more confident in their response when there was no click, *F*_(2, 21)_ = 7.66, *p* = 0.012, *n*^2^ = 0.267. There was, however, no significant influence of Note Type, *F*_(2, 42)_ = 0.79, *p* = 0.46 or Age Group, *F*_(1, 21)_ = 1.87, *p* = 0.19, on confidence during the click-detection task.

#### Pitch-detection task

**Figure 3:**
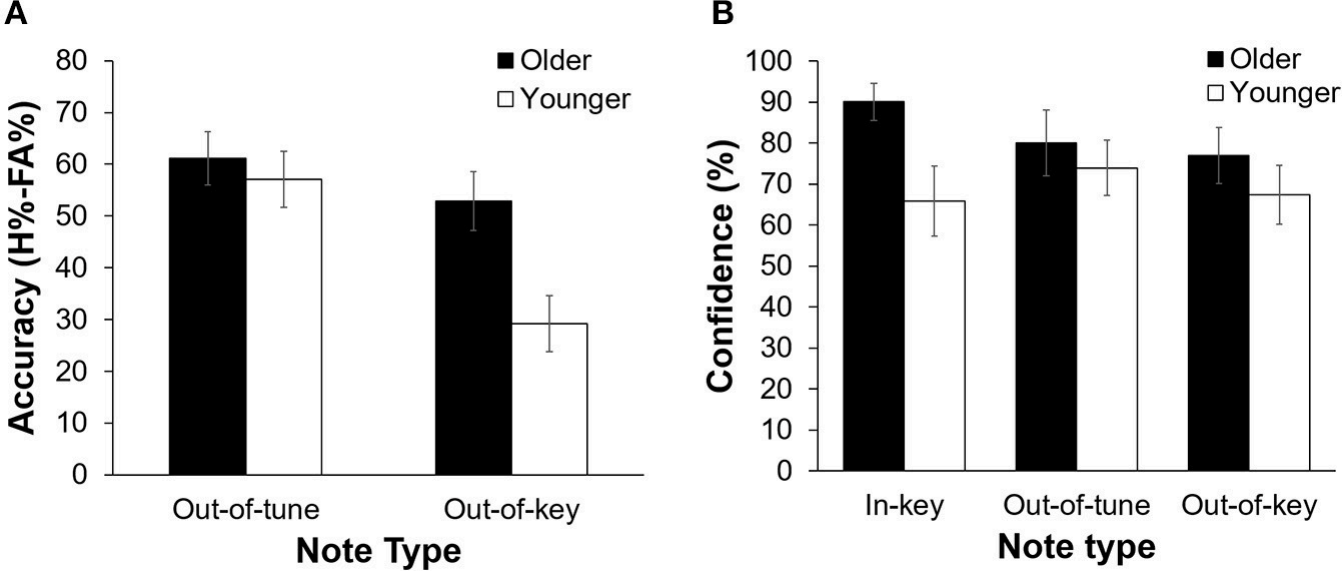
**(A)** Pitch-detection accuracy (H%–FA%) as a function of Age Group and Note Type. Overall, participants were better able to detect an out-of-tune note compared to an out-of-key note (*p* < 0.001). Older adults were better than younger adults at detecting an out-of-key note (*p* = 0.005). There was no significant difference in performance between groups for the in-key and out-of-tune notes. Errors bars represent one standard error of the mean. **(B)** Pitch-detection confidence (% of “sure” responses) as a function of Age Group and Note Type. Older adults were more confident than younger adults when identifying an in-key note (*p* = 0.024). Confidence did not differ between older and younger adults for the out-of-key or out-of-tune notes. Errors bars represent one standard error of the mean.

**Figure 4:**
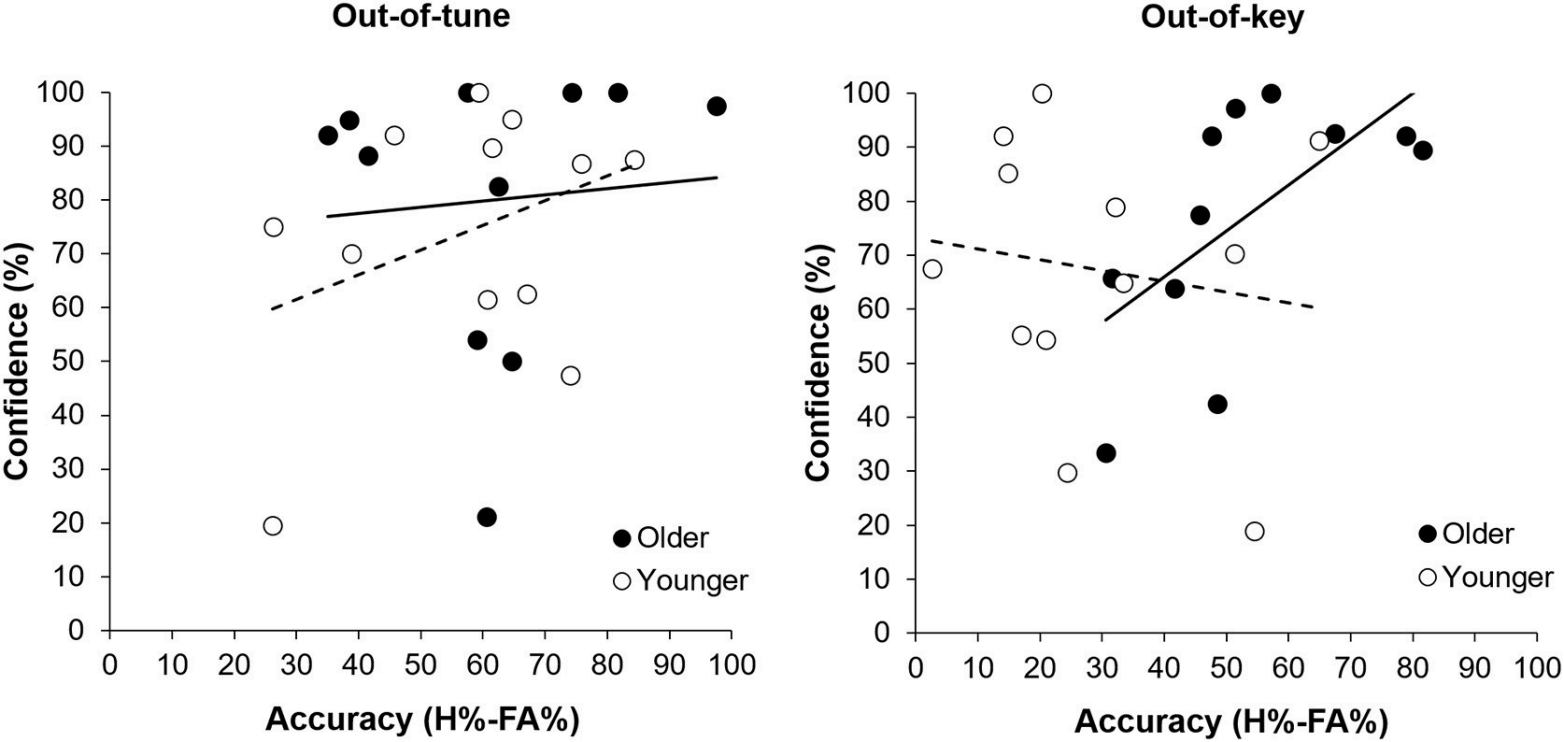
Accuracy (percentage hits minus false alarms) for detecting a melodic violation as a function of response confidence (percentage “sure” responses), separated by group. There was a positive correlation between response confidence and accuracy when an out-of-key note was presented for older adults (*p* = 0.035). No other correlation was significant.

Overall, participants were more accurate at detecting an out-of-tune note compared to an out-of-key note, *F*_(1, 21)_ = 26.39, *p* < 0.001, *n*^2^ = 0.557. Interestingly, the interaction between Age Group and Note Type was significant, *F*_(1, 21)_ = 7.80, *p* = 0.011, *n*^2^ = 0.271 (see Figure 3). Specifically, older adults were more accurate than younger adults at detecting out-of-key notes, *t*_(21)_ = 3.16, *p* = 0.005, but the groups did not differ in their ability to detect an out-of-tune note, *t*_(21)_ = 0.52, *p* = 0.61. There was also an interaction between Age Group and Note Type when looking at confidence scores, *F*_(2, 42)_ = 4.65, *p* = 0.025, *n*^2^ = 0.181. Pairwise comparisons revealed that both groups were similarly confident when detecting out-of-tune notes, *t*_(21)_ = 0.59, *p* = 0.56, and out-of-key notes, *t*_(21)_ = 0.96, *p* = 0.35, while older adults were more confident when an in-key melody was presented, compared to younger adults, *t*_(21)_ = 2.49, *p* = 0.024. Furthermore, accuracy when detecting an out-of-key note was positively correlated with confidence in the group of older adults, *r*_(9)_ = 0.64, *p* = 0.035. This was not the case for younger adults, *r*_(10)_ = −0.15, *p* = 0.64 (see Figure 4). In both groups, accuracy and confidence were not correlated for out-of-tune notes [*r*_(9)_ = 0.08, *p* = 0.81; *r*_(10)_ = 0.37, *p* = 0.23, for older and younger adults, respectively].

### Electrophysiological results

#### Click-detection task

##### Younger adults

**Figure 5:**
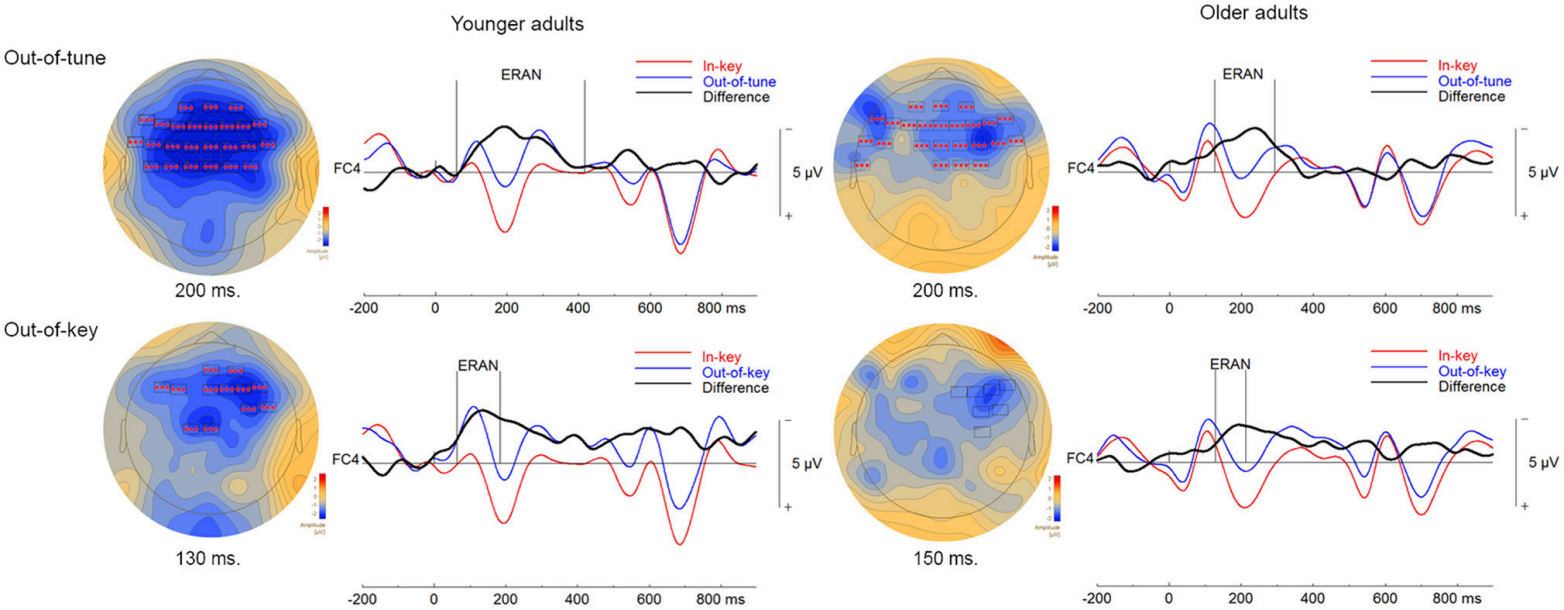
ERPs in the click-detection task. For the topographical plots, boxes represent electrodes included in the cluster. ERPs from electrode FC4 are plotted to the right of the topographies. Out-of-tune/out-of-key notes are presented in blue, in-key notes are presented in red, and their difference is presented in black. Additionally, vertical lines indicate the epoch of the cluster identified.

Three significant clusters were found when comparing ERPs to in-key and out-of-tune pitch conditions in younger adults during the click-detection task. For the first cluster, the ERP for the out-of-tune notes was more negative than for the in-key notes (*p* < 0.001). This cluster lasted from 59 to 418 ms and included electrodes widespread around the scalp. Considering its time window, this cluster, likely represents an ERAN (see Figure 5). The second cluster also indicated a more negative ERP response evoked by the out-of-tune tones at frontocentral electrodes, from 0 to 31 ms (*p* = 0.008). The third cluster again showed a more negative ERP response to out-of-tune notes between 516 and 552 ms at central and right frontocentral electrodes (*p* = 0.046).

Two significant clusters were identified when comparing out-of-key to in-key notes. For the first cluster, from 64 to185 ms, the ERP evoked by the out-of-key notes was more negative than the ERP evoked by the in-key notes at frontocentral electrodes (*p* < 0.001). Given its topography, this cluster is most likely an ERAN, although its latency is earlier than what is usually expected for an ERAN (shown in Figure 5). The second cluster indicated a more negative ERP response to out-of-key notes from 555 to 613 ms at right and central frontal electrodes (*p* = 0.028).

##### Older adults

One significant cluster was identified when comparing the ERP for the in-key and out-of-tune tones in older adults during the click-detection task. For this cluster, the ERP for the out-of-tune notes was more negative than for the in-key notes, mostly at frontocentral electrodes, and lasted from 125 to 293 ms (*p* < 0.001). With its latency and topography, this cluster corresponds to an ERAN.

When comparing the in-key and out-of-key notes, two significant clusters were identified. For the first cluster, the ERP for the out-of-key notes was more negative than for the in-key notes (*p* = 0.007). This cluster included electrodes widespread across the scalp and had a time window from 603 to 701 ms. The second cluster indicated an ERP for the out-of-key notes that was more negative than for the in-key notes (*p* = 0.016). The second cluster lasted from 705 to 798 ms and was located at posterior and frontocentral electrodes. A cluster was identified where the ERP for the out-of-key notes was more negative than the ERP for the in-key notes, from 129 to 213 ms at right frontocentral electrodes that might represent an ERAN. However, this cluster failed to reach significance (*p* = 0.20; shown in Figure 5).

#### Pitch-detection task

##### Younger adults

**Figure 6:**
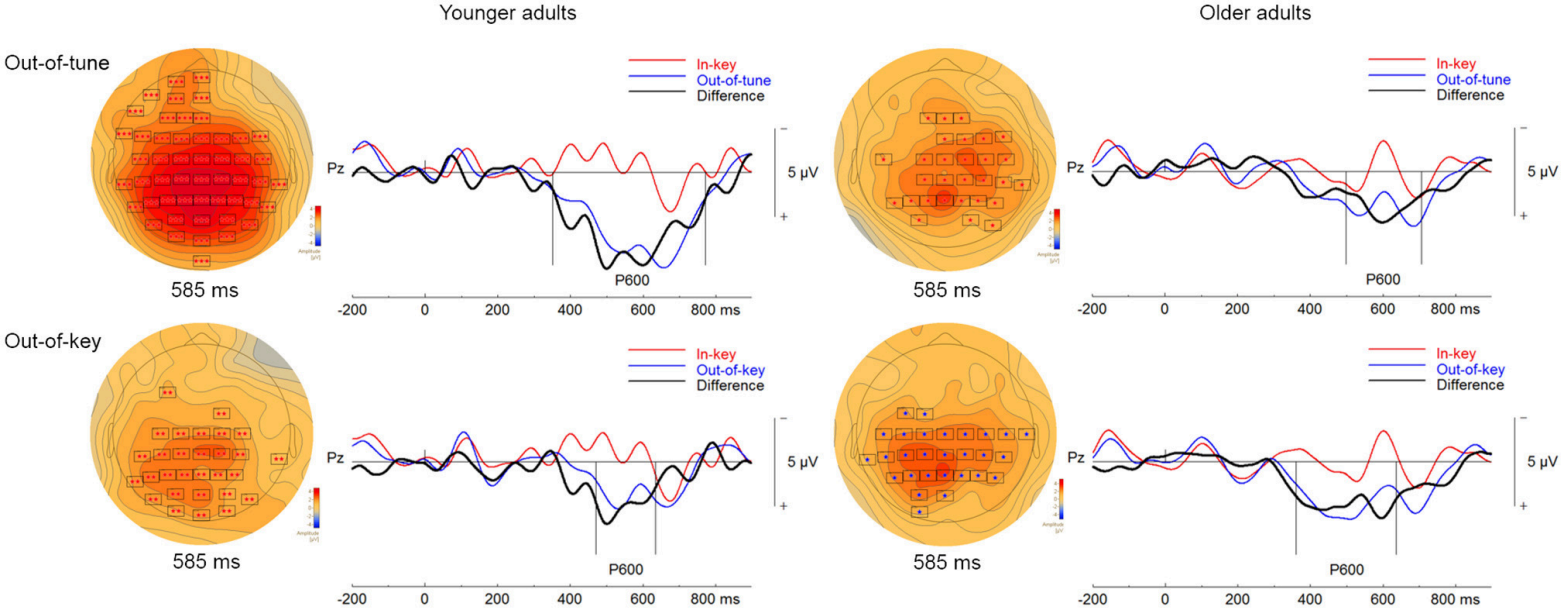
ERPs in the pitch-detection task. For the topographical plots, the boxes represent electrodes where the difference was significant at the reported time. ERPs from electrode Pz are plotted to the right. Out-of-tune/out-of-key notes are presented in blue, in-key notes are presented in red, and their difference is presented in black. Additionally, vertical lines indicate the epoch of the cluster.

When comparing ERP responses to out-of-tune and in-key notes in the pitch-detection task, one significant cluster was identified. This cluster showed a more positive evoked response, widespread around the scalp, for out-of-tune notes from 351 to 772 ms (*p* < 0.001). Given this latency and its positive amplitude, this cluster is likely a P600 (see Figure 6). In addition, a cluster was identified where the ERP for the out-of-tune notes was more negative than the ERP for the in-key note from 156 to 247 ms at frontocentral electrodes, however this cluster failed to reach significance (*p* = 0.12). This cluster likely reflects an ERAN.

There was also one significant cluster, shown in Figure 6, when comparing out-of-key and in-key notes. This cluster, including electrodes widely distributed around the scalp, indicated a more positive ERP response to the out-of-key notes than to the in-key notes (*p* = 0.007). The time window of this cluster was from 472 to 635 ms and thus is likely a P600. Only a small non-significant cluster (*p* = 0.27) was observed where there was an increased negativity for out-of-key notes compared to in-key notes. This cluster was however at left-frontal sites and occurred from 271 to 306 ms, thus no significant cluster seemed to correspond to an ERAN-like ERP.

##### Older adults

One significant cluster was identified when comparing ERPs to the in-key and out-of-tune notes in the group of older adults during the pitch-detection task. The ERP for the out-of-tune notes was more positive than for the in-key notes (*p* = 0.005). This cluster time window was from 498 to 707 ms and included right frontocentral and posterior electrodes. Given its latency and topography, this cluster most likely corresponds to a P600 (Figure 6). No other cluster was identified as an ERAN-like ERP. Although there was a cluster of right frontal electrodes, that failed to reach significance, where the ERP for the out-of-tune notes was more negative than for the in-key notes between 144 and 191 ms (*p* = 0.39).

Two significant clusters were identified when comparing evoked response to in-key and out-of-key notes. For the first cluster, the ERP for the out-of-key notes was more positive than for the in-key notes, from 361 to 546 ms at electrodes widespread around the scalp (*p* = 0.001). This first cluster likely corresponds to a P600. For the second cluster, the ERP for the out-of-key notes was also more positive than for the in-key notes (*p* = 0.007). This cluster was present from 555 to 637 ms at central and posterior electrodes, and thus might be a continuation of the P600. Both clusters are represented in Figure 6. No significant cluster seemed to identify an ERAN-like ERP. There was a cluster of right centroparietal electrodes from 271 to 306 ms, where the ERP to out-of-key notes was more negative than the response elicited by in-key notes which failed to reach significance (*p* = 0.28).

#### Group differences

**Figure 7:**
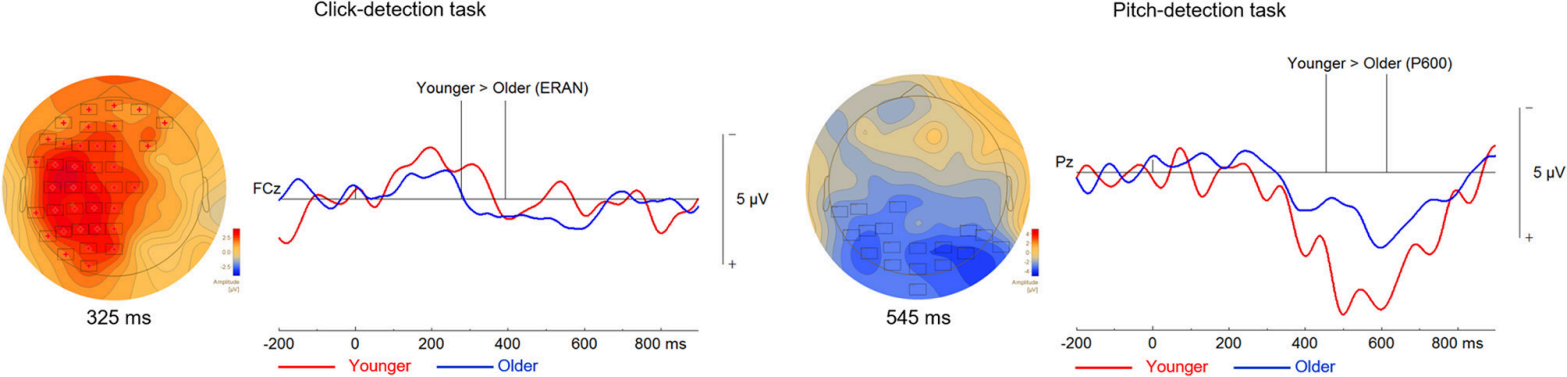
Differences between the ERPs of younger and older adults during the click-detection task to the left and the pitch-detection task to the right. For all topographical plots, the boxes represent electrodes where the difference was significant at the reported time. Difference waves (out-of-tune minus in-key) are presented to the right of the topographical maps, at electrode FCz for the click-detection task and Pz for the pitch-detection task. For these ERPs plots, the group of older adults is presented in blue and the group of younger adults is presented in red. Additionally, vertical lines indicate the epoch when the difference was significant.

We compared the ERAN response of younger and older adults evoked by the out-of-tune and out-of-key notes in the click-detection task (depicted in Figure 7). We choose individual time windows for each EPR that corresponded to the earliest and latest latency identified in both groups for each ERP. For the out-of-tune notes, the time window tested was from 59 to 418 ms. It was found that the ERAN response was larger in younger adults (*p* = 0.049). For out-of-key notes, using a time window of 64 to 213 ms, the permutation tests revealed no significant clusters (*p* > 0.15 for all clusters). For the pitch-detection task, we focused on the P600, since a reliable ERAN was not observed in either group for either the out-of-tune or out-of-key notes. For the out-of-tune notes, the P600 was larger in younger adults compared to older adults at central and parietal electrodes during the 454–614 ms epoch (*p* = 0.05). For the out-of-key notes, none of the permutations revealed a significant cluster during the 361 to 637 ms epoch (*p* > 0.4 for all clusters).

### Relationship between ERPs responses and behavior

To evaluate the relationship between the accuracy at detecting the incongruity (i.e., H-FAs), the ERAN evoked by pitch incongruity in the click-detection task, and the P600 evoked by the pitch incongruity during the pitch-detection, a multiple regression was performed across participants. The mean amplitude of the ERAN at FC4 and the P600 at Pz (using the difference waves) were entered as independent factors in the model as predictors of H-FA scores. These electrodes were selected because they formed part of the ERAN/P600 cluster for all participants, and because previous research identified that these electrodes provide a good estimate of the ERAN and P600. Mean amplitude was calculated using the epoch defined by the permutation testing in the previous section.

**Figure 8:**
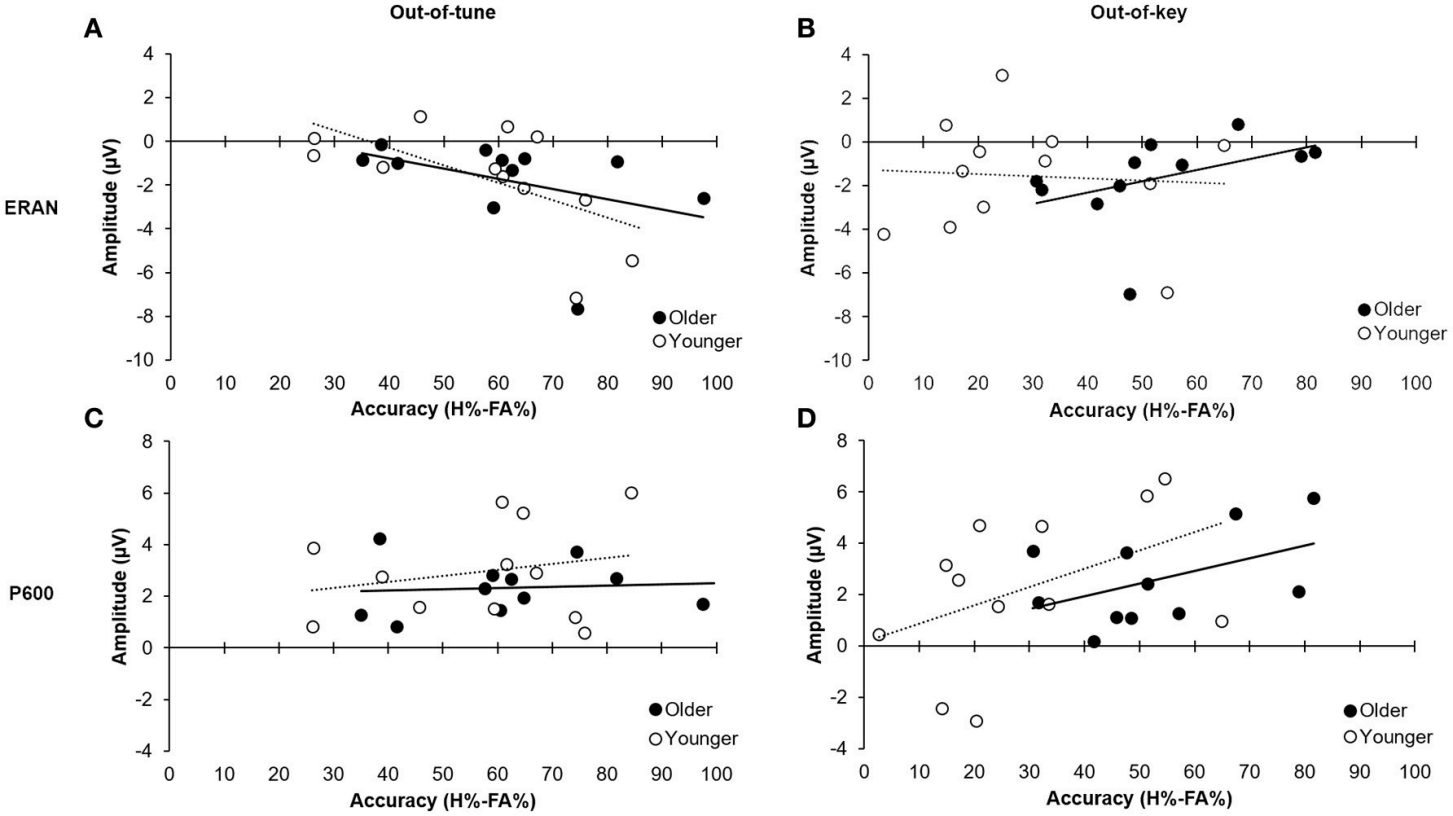
Relationship between ERP mean amplitude and accuracy (H%–FA%). **(A)** Relationship between amplitude of the ERAN for the out-of-tune notes in the click-detection task and detection accuracy for out-of-tune notes in the pitch-detection task. The correlation was significant when including all participants (*p* = 0.01); however, the effect was stronger in the younger adults compared to the older adults. **(B)** Relationship between the amplitude of the ERAN for the out-of-key notes in the click-detection task and the detection accuracy for the out-of-key notes during the pitch-detection task. The correlation was not significant between the two variables. **(C)** Relationship between the amplitude of the P600 for the out-of-tune notes in the pitch-detection task and accuracy. There was no significant correlation between these two variables. **(D)** Relationship between amplitude of the P600 for the out-of-key notes in the pitch-detection task and the accuracy to detect these. There was a moderate correlation when considering all participants (*p* = 0.05), and the effect was similar in both older and younger participants.

For the out-of-tune notes, the overall model was significant, *F*_(2, 20)_ = 3.84, *p* = 0.04, *adj.R*^2^ = 0.21. The amplitude of the ERAN explained most of the variance in the model [β = −0.513, *t*_(20)_ = −2.67, *p* = 0.02], while the P600 did not explain any additional variance in H-FA [β = 0.062, *t*_(20)_ = 0.32, *p* = 0.75]. Along these lines, there was a strong correlation between the ERAN for the out-of-tune notes in the click-detection task and the accuracy to detect this type of incongruity during the pitch-detection task, when considering all participants, *r*_(21)_ = −0.52, *p* = 0.01. However, this correlation was stronger in the younger group, *r*_(10)_ = −0.61, *p* = 0.03, than for older participants, *r*_(9)_ = −0.41, *p* = 0.20 (see Figures 8A,C). Given that the ERAN-Accuracy correlation was weaker in older adults, an additional exploratory analysis was carried out to test the idea that detection of an out-of-tune note might be related to enhanced neural activity over frontal regions during the pitch-detection task. Accordingly, correlations were calculated between P600 amplitude at electrode Fz and Accuracy (H-FA) for the out-of-tune note in older and younger adults. Neither correlation was significant, however the correlation coefficient in older adults, *r*_(9)_ = −0.39, *p* = 0.23, was much larger than for younger adults, *r*_(10)_ = −0.07, *p* = 0.83. The negative correlation suggests that the emergence of a frontal N600 (i.e., a polarity reversal of the P600) may contribute to the ability to detect an out-of-tune note in older adults.

For the out-of-key notes, the overall multiple regression model was marginally significant, *F*_(2, 20)_ = 2.86, *p* = 0.081, adj.R2 = 0.14, and mostly due to the relationship between the P600 amplitude and H-FA [β = 0.488, *t*_(20)_ = 2.34, *p* = 0.030]. The ERAN did not significantly contribute to the variance of H-FA scores [β = 0.252, *t*_(20)_ = 1.21, *p* = 0.24]. A moderate positive correlation, between the P600 amplitude and accuracy to detect the out-of-key notes, suggests that with bigger P600 amplitude we could expect better H-FA scores [*r*_(21)_ = 0.41, *p* = 0.05 across participants; *r*_(10)_ = 0.45, *p* = 0.14 and *r*_(9)_ = 0.47, *p* = 0.14 for young and older adults, respectively; see Figures 8B,D).

## Discussion

The aim of this study was to examine how processing of musical tonal incongruities differs between older and younger adults. To do so we compared the ERAN and P600 evoked by both an out-of-key and an out-of-tune note between a group of younger and older adults. A click-detection and pitch-detection task were used to further isolate the impact of attention directed toward the melodic incongruities, and thus dissociate automatic and cognitive/controlled processing of tonality. In both groups, a clear ERAN was evoked by both types of tonal incongruities in the click-detection task, and a P600 was evoked by both types of tonal incongruities in the pitch-detection task. This pattern of evoked responses is consistent with previous research that has used similar paradigms (Brattico et al., 2006; Zendel et al., 2015). No clear ERAN was found in the pitch-detection task contrary to what could have been expected. Its absence in this study is likely due to the permutation testing used to evaluate statistical significance which is data driven and rather conservative. A visual inspection of the data suggests however that there was a small ERAN in the pitch-detection task.

Comparisons between groups during the pitch-detection task indicated that older adults were better able to detect an out-of-key note compared to younger adults, but the ERAN and P600 associated to this type of deviant were comparable in both age groups. For the out-of-tune notes, there was no difference between the groups in the ability to detect a deviant note, while the ERAN and P600 were reduced in older adults compared to younger adults. Overall, these results confirm our general prediction that processing of out-of-key notes would be enhanced in older adults compared to younger adults, while processing out-of-tune notes would be similar in older adults compared to younger adults. The specific pattern of findings also suggests that older adults may engage alternative cognitive mechanisms to make tonal judgments.

A recent model introduced by a group of hearing scientists (Pichora-Fuller et al., 2016) explains how aging impacts hearing based on two types of attention related processes. This model extends the Kahneman (1973) *capacity of attention* model. Pichora-Fuller et al. (2016) propose that attention is allocated to a hearing task in two ways: automatically and intentionally; these are based on Kahneman (1973) terms, “enduring dispositions” and “momentary intentions.” Automatic attention (enduring dispositions) refers to the automatic orientation of attention to an unexpected sound in the environment. Intentional attention (momentary intentions) refers to the process of intentionally allocating attention toward a specific auditory stimulus. According to this model, older adults tend to exert more intentional attention toward the auditory environment to overcome age-related decline in hearing abilities. In the context of this study, the ERAN evoked in the passive listening task likely reflects automatic allocation of attention, while the P600 response likely represents the intentional recruitment of cognitive resources to detect and incorporate the tonal incongruity into the current melodic context. Across-participants, both types of incongruities elicited an ERAN and a P600 response. However, the association between these responses and the type of deviant (i.e., out-of-tune or out-of-key) differed between age groups.

The amplitude of the ERAN in the click-detection task predicted the ability to later detect out-of-tune notes in the pitch-detection task, but not the out-of-key notes, although this relationship was stronger in the younger adults. This suggests that out-of-tune notes can be detected based on the automatic attentional system, as the performance of this system (as indexed by the ERAN) was related to task-performance. Moreover, since out-of-tune notes are based on a violation of a lower level of the tonal hierarchy (i.e., the chromatic scale), they are more unexpected and thus evoke a larger automatic response. Further support for this proposal comes from the click-detection task, where all participants experienced more interference from the out-of-tune notes compared to both the out-of-key notes and the in-key notes. That is, the ability to detect a click was reduced when there was an out-of-tune note in the melody compared to when the melody was in-key or contained an out-of-key note. These findings support previous work showing that out-of-tune notes are more perceptually salient than out-of-key notes (Brattico et al., 2006). Although older adults had a reduced ERAN evoked by out-of-tune notes, they detected these notes with the same accuracy as younger adults. The out-of-tune notes also had a similar interfering effect on the click-detection task in both age-groups. Thus, it might be possible that older adults used additional cognitive processes when detecting the out-of-tune notes, which would explain why the relationship between the ERAN and accuracy for out-of-tune notes was stronger in the group of younger adults compared to the older adults. A previous study suggested that older adults exhibit a decreased automatic response to an auditory deviant but an increased intentional/controlled response to an auditory deviant (Alain et al., 2004). Specifically, when deviant stimuli were individually calibrated to be equally perceivable by both younger and older adults, the MMN was reduced in amplitude in older adults compared to younger adults, while the P3 response was similar in amplitude in both groups. This result was interpreted by the authors as an increased reliance on cognitive mechanisms to overcome deficits in the encoding of acoustic information with aging. Here, the saliency of tonal deviant could not be individually calibrated *per se* because it needed to fit musical theory but similarly, task difficulty was controlled between participants by asking them to detect an individually calibrated near-threshold click. In that respect, our results are consistent with the proposal that older adults are using additional cognitive resources, beyond the automatic attentional system, to detect an out-of-tune note that deviates from the chromatic scale.

Interestingly, during the pitch-detection task, the P600 evoked by an out-of-tune note was also reduced in older adults compared to younger adults; however, the reduction was mainly at posterior sites, suggesting that frontal activity during this time frame may be related to the similarity in accuracy between younger and older adults. Indeed, there was a stronger relationship between brain activity evoked by an out-of-tune note at a frontal electrode and accuracy when detecting an out-of-tune note for older adults compared to younger adults. Moreover, this relationship was negative, further indicating that the benefit may be related to a small frontal N600. This frontal N600 may be a source of compensatory activity in the group of older adults as it was only evident during the active task. The relationship between the ERAN evoked during the click-detection task and accuracy when detecting an out-of-tune note was weaker in older adults compared to younger adults. Taken together, this pattern of results suggests that older adults are increasingly relying on cognitive mechanisms supported by frontal regions to process out-of-tune notes. This shift is likely due to age-related changes in the automatic processing of tonal deviants. The current finding is consistent with studies of syntactic processing of speech in older adults that shows a shift toward frontal electrodes in the topography of the P600, without a change to P600 amplitude (Steinhauer et al., 2010). These results could also be compatible with the posterior-anterior shift framework in cerebral activations with aging (Davis et al., 2007). According to this framework, older adults would optimize the use of knowledge to make predictions, through top-down process, rather than relying mostly on online sensory information, which would be best reflected by greater activation of frontal regions and/or a stronger relationship of frontal regions to task performance (Davis et al., 2007; Moran et al., 2014).

An unexpected, but related finding was the observation of a late negativity over right fronto-central electrodes during the click-detection task. In young adults this was observed for both out-of-key and out-of-tune notes between 500–600 ms after the tonal violation. In older adults this effect was only observed for the out-of-key notes. In older adults the activity was more widespread around the scalp, was delayed, and was longer (700–900 ms). The auditory N600 has sources in the inferior frontal gyrus (IFG; Shahin et al., 2006). The right IFG is a region that is critical for processing tonal structure in music, and is known to become active when there are tonal violations (Zatorre et al., 1994; Tillmann et al., 2003). This N600 could reflect an automatic activation of this structure, and the lack of N600 during the pitch detection task is likely due to the P600 which is more dominant during this epoch. The finding that the N600 during the click-detection task was delayed in older adults when presented with an out-of-key note, and not observed when presented with an out-of-tune note suggests a reduction in automatic processing of tonal violations in older adults. When paired with the finding that a frontal negativity predicted task performance for detecting the out-of-tune notes during the pitch-detection task, this pattern of results further supports the idea that there is decline in automatic processing of tonal information that is compensated for by frontal attentional mechanisms.

Out-of-key notes were harder to detect and did not impact the ability to detect a click in both older and younger adults. Older adults were, however, better at detecting the out-of-key notes. Still, there were no group differences in the ERAN or P600 amplitude. A very recent study examined electrophysiological responses to the final note in a short melody that differed in terms of its musical expectancy (but was in-key) and found no differences between older and younger adults in their ratings (good—bad) of these melodies (Halpern et al., 2017). At the same time unexpected melodic terminations evoked ERAN- and P600-type responses in both age groups, consistent with the current findings (Halpern et al., 2017). Moreover, Halpern et al. (2017) reported a general age effect across both expected and unexpected endings during the P600 time window, suggesting that older adults are deploying more controlled attentional resources to melodic processing. This could explain why, in the present study, the older adults were better able to detect out-of-key notes, without specific effects on the ERAN or P600. The detection of an out-of-key note necessitates the integration of the violation in terms of the tonal context which would require an additional processing stage that is likely intentional.

Intentional processing of melodic information in older adults may be facilitated by enhanced crystalized knowledge of Western tonal structure due to increased hours of musical exposure throughout life. Crystalized knowledge is the result of previous cognitive processing, such as general knowledge or vocabulary. One of the current theories related to aging is that tasks that rely on basic information processing (fluid intelligence) tend to decline in older adults, while tasks that rely on crystalized knowledge tend to be preserved (Lindenberger et al., 2001; Salthouse, 2011; Harada et al., 2013). In the current study, it is reasonable to assume that detection of an out-of-key note required a more robust representation of tonality compared to the detection of an out-of-tune note (Brattico et al., 2006). Since all participants were non-musicians, it is likely that the older participants have accumulated more time listening to music throughout their lives compared to the younger adults (note: we did not quantify hours of lifetime music exposure, we are making the assumption that older and younger adults were exposed to a similar amount of music per year of life, and because of that, older adults have more musical exposure). This long-term exposure would strengthen the representation of tonality, facilitating the ability to detect out-of-key notes. This is also supported by our finding that the accuracy of older adults when detecting an out-of-key note was related to the confidence of their response. The correlation between confidence and accuracy could reflect an enhanced awareness of the tonal violation in older adults (Maniscalco and Lau, 2012; Fleming and Lau, 2014). Enhanced awareness of the tonal violation would be reflected in the recruitment of the frontal regions, as these regions are essential to self-awareness, or in other words, the integration of past experience in guiding behavioral response (Stuss and Alexander, 2000). Indeed, a number of studies have shown that when older adults perform a task they tend to over-engage frontal regions compared to younger adults (Gutchess et al., 2005; Reuter-Lorenz and Lustig, 2005; Mattay et al., 2006). This general pattern reflects a posterior-anterior shift when performing cognitive tasks (Davis et al., 2007).

Although it is likely that older adults have more total hours of musical exposure, both younger and older adults are “overexposed” to music. If we assume that the ability to have “learned” a music system (based on passive exposure via statistical learning) reaches a maxima after a few years of exposure, then differences in the ability to detect an out-of-key note could be due to differences in music style exposure, that is a cohort effect. Most people tend to listen to similar music throughout life and the type of music older adults listen to is quite different than the music listened to by younger adults (Smith, 1994; Harrison and Ryan, 2010; Bennett and Taylor, 2012; Bonneville-Roussy et al., 2013). With the internet, it is now possible to access myriad musical genres and younger adults are much more likely to use Internet music services (Glévarec and Pinet, 2012). Critically, some of these genres might not necessarily follow the rules of Western tonality. It is therefore possible that younger adults are more “liberal” in their judgment of out-of-key notes, by having a less restrained representation of tonality. This would explain why the older adults were better able to detect an out-of-key note compared to younger adults. That is, older adults have more exclusive exposure to music resembling the type of music used in this experiment. In both cases, these interpretations are consistent with the idea that crystalized knowledge of tonal structure is enhanced in older adults and this knowledge could support top-down processes that allow the auditory system to form more accurate and robust predictions of upcoming tones in a melody.

Future studies should assess the contribution of tonal knowledge and musical background on the neurophysiological response to tonal deviants. Although in the current study musical training was taken into account in participants' selection, details on music listening habits and musical preferences could have provided a more robust estimate of Western tonal knowledge or exposure. Comparisons of older and younger musicians and non-musicians could help to address this limitation. Further, while cross-sectional studies provide insights on age-related changes, it is important to recognize that differences between older and younger adults are not necessarily effects of aging. A longitudinal study would allow for stronger claims on the idea of compensation mechanisms. Indeed, a longitudinal approach would enable the observation of how age-related changes in evoked responses to tonal incongruities are related to differences in baseline abilities and exposure to music.

Data from the current study are consistent with the idea that there is an age-related increase in crystalized knowledge about the tonal structure of music. Increased knowledge of tonality could additionally compensate for age-related decline in central auditory processing. This would allow for music perception to be generally preserved in older adults, despite neurophysiological changes in how music is processed. That is, access to crystalized knowledge could enhanced perception or predictability of complex auditory stimuli in older adults.

## Author contributions

ML, IP, and BZ: contributed to design of the study; ML and BZ: did the data analysis; ML wrote the manuscript, with feedback from IP and BZ.

### Conflict of interest statement

The authors declare that the research was conducted in the absence of any commercial or financial relationships that could be construed as a potential conflict of interest.
